# Case Report: Monochorionic triamniotic triplet pregnancy following single vitrified-warmed blastocyst transfer

**DOI:** 10.3389/frph.2026.1977805

**Published:** 2026-10-12

**Authors:** Artem A. Aksenenko, Tatyana A. Kodyleva, Polina O. Patskova, Alla A. Gavisova, Gennadiy T. Sukhikh

**Affiliations:** 1 Department of Preservation and Restoration of Reproductive Function, National Medical Research Center for Obstetrics, Gynecology and Perinatology named after Academician V.I. Kulakov, Ministry of Health of the Russian Federation, Moscow, Russia; 2 National Medical Research Center for Obstetrics, Gynecology and Perinatology named after Academician V.I. Kulakov, Ministry of Health of the Russian Federation, Moscow, Russia

**Keywords:** assisted reproductive technology, blastocyst transfer, embryo splitting, monochorionic triamniotic triplet, monozygotic triplet, single embryo transfer, vitrification

## Abstract

Monochorionic triamniotic triplet pregnancy following single embryo transfer is an exceptionally rare outcome of assisted reproductive technology. We report a monochorionic triamniotic triplet pregnancy following transfer of a single vitrified-warmed blastocyst. Fertilization was performed by intracytoplasmic sperm injection, with no assisted hatching, trophectoderm biopsy or preimplantation genetic testing. Initial transvaginal ultrasound 21 days after embryo transfer demonstrated a singleton intrauterine pregnancy. At 8 weeks and 1 day of gestation, three viable embryos were identified within a monochorionic triamniotic pregnancy. The pregnancy remained uncomplicated, with appropriate growth of all three fetuses, and three live female infants were delivered at 35 weeks of gestation. This case adds to the limited number of reported monochorionic triamniotic triplet pregnancies following single embryo transfer and is distinguished by transfer of a day-6 vitrified-warmed blastocyst that re-expanded from grade 2BB after warming to 6BB before transfer, without assisted hatching, trophectoderm biopsy or preimplantation genetic testing.

## Introduction

Single embryo transfer (SET) is the principal and preferred strategy for reducing iatrogenic multiple pregnancy in assisted reproductive technology (ART) and for maximizing the likelihood of a singleton pregnancy. Current ESHRE and ASRM recommendations support limiting the number of embryos transferred and favor SET in patients in whom transfer of more than one embryo would provide little additional benefit while increasing the risk of multiple gestation (1–3). A small residual risk of multiple pregnancy remains after SET because a transferred embryo may undergo monozygotic splitting.

**Figure 1:**
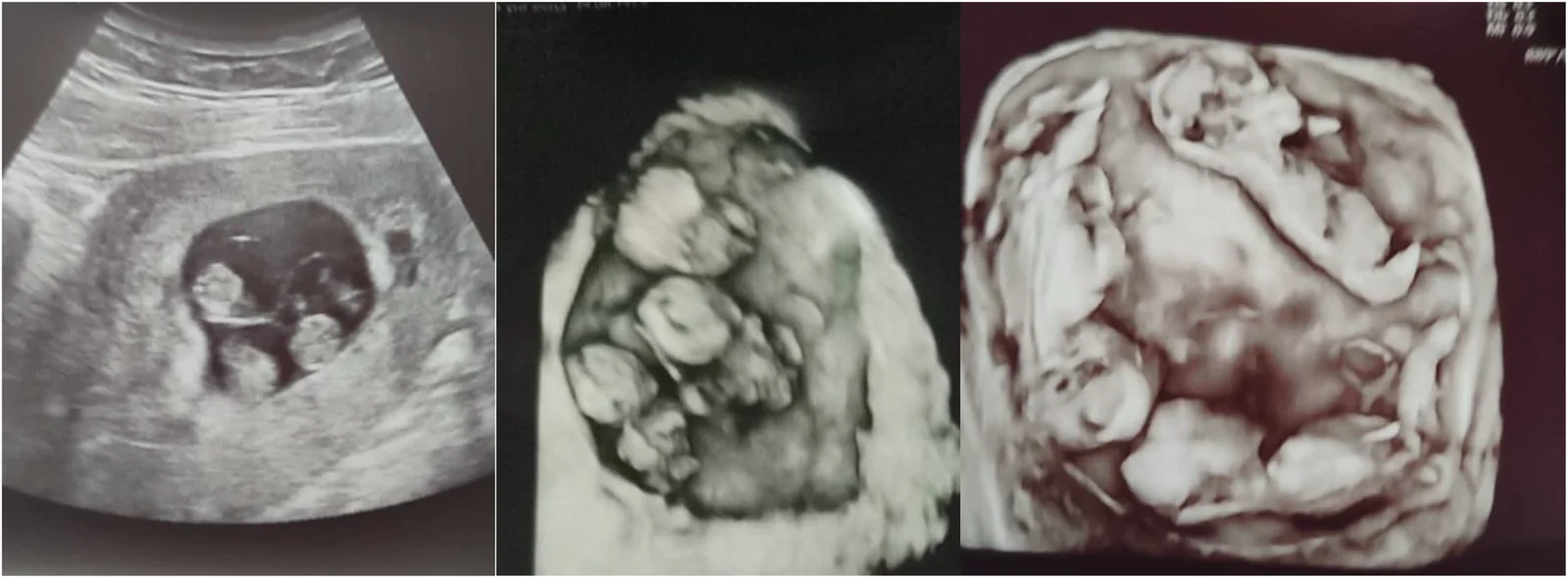
Ultrasound examination at 8 weeks of gestation demonstrating a monochorionic triamniotic triplet pregnancy.

The residual multiple-pregnancy risk after SET predominantly reflects monozygotic twinning. In a retrospective cohort of 28,596 pregnancies following fresh, non-donor SET, Kanter et al. reported monozygotic twinning rates of 1.71% after day 2–3 transfer and 2.50% after day 5–6 transfer (4). Monozygotic triplet pregnancy is substantially rarer. A prevalence of approximately 0.04%–0.05% has been reported in large ART datasets, although estimates are based on very small numbers of events and should be interpreted cautiously (5). Only a small number of monozygotic triplet pregnancies following transfer of a single embryo have been described in the literature (Table 1) (6–11).

**Table 1:** Reported clinical cases of triplet pregnancy following single embryo transfer.

Author, year	Age	Embryo/day/morphology	ART characteristics	Outcome
Rísquez et al., 2004	40	Day 3, fresh transfer; grade NR	ICSI+assisted hatching	Ongoing pregnancy (at the time of publication)
Faraj et al., 2008	38	Day 5, frozen-thawed transfer; grade NR	Donor oocytes	Cesarean delivery at 32 weeks; live birth
Lee et al., 2008	28	Day 5, fresh transfer; grade NR	ICSI	Cesarean delivery at 33 weeks; live birth
Ferri et al., 2020	43	Day 5, fresh transfer; grade NR	Donor oocytes	Nonviable pregnancy
Ota et al., 2020	32	Day 5, frozen-thawed; 4BC	IVM+ICSI	Cesarean delivery at 33 weeks; live birth
Woods et al., 2025 (case 1)	29	Day 5, frozen-thawed transfer; grade NR	ICSI+PGT-A + embryo reduction	Delivery at 36 weeks; live birth (1 infant)
Woods et al., 2025 (case 2)	34	Day 5, frozen-thawed transfer; grade NR	ICSI	Cesarean delivery at 35 weeks; live birth
Present case	39	Day 6, vitrified-warmed; 2BB after warming → 6BB at transfer	ICSI; no AH, TE biopsy or PGT	Cesarean delivery at 35 weeks; 3 live births

The biological mechanisms responsible for monozygotic embryo splitting remain incompletely understood. Blastocyst-stage transfer, assisted hatching, embryo cryopreservation, intracytoplasmic sperm injection and trophectoderm biopsy have all been investigated as potential contributing factors, but no single ART-related procedure has been consistently identified as an independent cause of monozygotic splitting (5, 12, 13). We report a monochorionic triamniotic triplet pregnancy resulting in three live births following transfer of a single vitrified-warmed blastocyst without assisted hatching or trophectoderm biopsy. This case provides a well-documented clinical and embryological context in which to consider the proposed mechanisms of monozygotic triplet formation.

## Case report

### Patient history and previous ART treatment

A 39-year-old woman presented to the National Medical Research Center for Obstetrics, Gynecology and Perinatology named after Academician V.I. Kulakov, Ministry of Health of the Russian Federation in December 2018 with a 10-year history of infertility. She had previously undergone one unsuccessful ART cycle in 2014 using a gonadotrophin-releasing hormone (GnRH) antagonist protocol. Two metaphase II (MII) oocytes were retrieved, and two cleavage-stage embryos were transferred on day 3 without achieving pregnancy. In 2017, she underwent laparoscopic excision of bilateral ovarian endometriomas and adhesiolysis.

Evaluation at our center revealed an incomplete uterine septum, a left pelvic serocele measuring 5.0 × 3.5 cm, two left ovarian endometriomas measuring 1.5 and 1.8 cm, and findings consistent with peritoneal and left tubal endometriosis. Serum anti-Müllerian hormone concentration was 1.80 ng/mL. Semen analysis of the patient’s partner showed oligoasthenoteratozoospermia, with a sperm concentration of 7 × 10⁶/mL, progressive motility of 20%, and 1% morphologically normal forms.

### Index ART cycle and embryo development

In April 2019, controlled ovarian stimulation was performed using a GnRH antagonist protocol. Eight MII oocytes were retrieved and fertilized by intracytoplasmic sperm injection (ICSI). Embryos were cultured under reduced oxygen conditions (5% O₂) to the blastocyst stage. Embryo development was assessed on days 3 and 5, and blastocysts were graded according to the ESHRE/ALPHA morphological assessment criteria (14).

Two blastocysts were suitable for vitrification: a 3AB blastocyst on day 5 and a 2BB blastocyst on day 6. Vitrification was performed using Kitazato media (Japan) according to the manufacturer’s instructions.

Because surgical treatment of the uterine and pelvic pathology was planned before embryo transfer, both blastocysts were cryopreserved. In July 2019, the patient underwent laparoscopic adhesiolysis, fenestration and drainage of the pelvic serocele, left ovarian resection and coagulation of endometriotic lesions. Hysteroscopic resection of the incomplete uterine septum and intrauterine adhesions was performed during the same treatment period.

### First frozen embryo transfer

In October 2019, the day-5 3AB blastocyst was warmed and transferred in a natural-cycle frozen embryo transfer. Micronized progesterone was administered at a dose of 600 mg/day, and embryo transfer was performed on the sixth day of progesterone administration. The transfer resulted in a singleton pregnancy. In July 2020, spontaneous labor occurred at 39 weeks of gestation, resulting in the vaginal delivery of a healthy male infant weighing 3800 g and measuring 54 cm, with Apgar scores of 8/9. The day-6 2BB blastocyst remained cryopreserved.

### Second frozen embryo transfer

In June 2022, the patient returned for transfer of the remaining cryopreserved blastocyst. Warming was performed using Kitazato media according to the manufacturer’s instructions. Immediately after warming, the blastocyst was graded as 2BB. Following short-term culture in G2 medium (Vitrolife, Sweden), the blastocyst re-expanded and was graded as 6BB at the time of transfer according to the ESHRE/ALPHA morphological assessment criteria (14).

No assisted hatching, trophectoderm biopsy or preimplantation genetic testing was performed. A single vitrified-warmed blastocyst was transferred in a natural-cycle frozen embryo transfer on the sixth day of micronized progesterone administration at a dose of 600 mg/day. Endometrial thickness on the day of embryo transfer was 9 mm.

### Pregnancy and neonatal outcome

Serum *β*-human chorionic gonadotrophin (*β*-hCG) concentrations were 104.6 IU/l on day 10 and 1912 IU/l on day 17 after embryo transfer. The first ultrasound examination, performed 21 days after transfer, demonstrated a singleton intrauterine pregnancy.

At 8 weeks and 1 day of gestation, ultrasound examination identified three viable embryos (Figure 1). Crown–rump length was 17 mm in all three embryos, with heart rates of 162, 168 and 166 beats/min, respectively. A monochorionic triamniotic triplet pregnancy was diagnosed. The diagnosis was confirmed by repeat ultrasound examination at 14 weeks of gestation.

The pregnancy progressed without major maternal or fetal complications. Prenatal screening examinations were performed at the recommended gestational ages and revealed no abnormalities. Cervical length remained within the normal range throughout pregnancy, with no evidence of cervical insufficiency. Serial ultrasound examinations demonstrated appropriate growth of all three fetuses, without fetal growth restriction or abnormal feto-placental blood flow. No clinically or laboratory significant maternal abnormalities were identified during pregnancy.

The patient was delivered by Caesarean section at 35 weeks of gestation. Three live female infants were born. The first infant weighed 2,200 g and measured 47 cm, with Apgar scores of 8/9; the second weighed 2,200 g and measured 46 cm, with Apgar scores of 8/9; and the third weighed 2,170 g and measured 44 cm, with Apgar scores of 8/8.

### Ethics and patient consent

Written informed consent for publication of the clinical information and de-identified images included in this case report was obtained from the patient.

## Discussion

This case describes a monochorionic triamniotic triplet pregnancy resulting in three live births after transfer of a single vitrified-warmed blastocyst. Seven comparable triplet pregnancies following single embryo transfer are summarized in Table 1 (6–11). The present case is distinguished by transfer of a day-6 blastocyst that was graded 2BB immediately after warming and 6BB at the time of transfer, indicating complete re-expansion and hatching during post-warming culture. No assisted hatching, trophectoderm biopsy or preimplantation genetic testing was performed.

The previously reported cases are embryologically heterogeneous (6–11). Rísquez et al. described triplets after transfer of a fresh day-3 embryo following ICSI and assisted hatching (6). The remaining reports involved day-5 blastocysts: Faraj et al. reported a frozen-thawed embryo derived from donor oocytes (7); Lee et al. reported a fresh blastocyst after ICSI (8); Ferri et al. reported a fresh blastocyst derived from donor oocytes (9); Ota et al. reported a vitrified-warmed 4BC blastocyst after IVM and ICSI (10); and Woods et al. described two frozen-thawed day-5 blastocyst transfers, one involving a euploid embryo after PGT-A and the other an untested embryo after ICSI (11). In contrast, the present pregnancy followed transfer of a day-6 vitrified-warmed blastocyst without assisted hatching, trophectoderm biopsy or PGT. This heterogeneity does not support any single ART procedure as a necessary condition for monozygotic triplet formation and is more consistent with a multifactorial process (5, 13).

The day-6 development of the transferred blastocyst is also relevant to the discussion of extended embryo culture. Blastocyst-stage transfer has been associated with a higher rate of monozygotic twinning than cleavage-stage transfer in some observational studies (4, 13). However, these data generally group day-5 and day-6 blastocysts together and do not establish delayed blastulation to day 6 as an independent risk factor. All previously reported blastocyst cases summarized in Table 1 involved day-5 embryos (7–11), making the day-6 blastocyst a distinguishing feature of the present case. Its causal relevance to embryo splitting remains unknown and should not be inferred from a single observation.

The traditional model of monozygotic twinning, originally proposed by Corner, relates chorionicity and amnionicity to the timing of embryonic splitting. This model has increasingly been questioned because some observations following ART cannot be readily reconciled with a simple temporal relationship between splitting and extraembryonic membrane formation (13). In particular, monozygotic pregnancies with unexpected patterns of chorionicity after transfer of a single blastocyst suggest that embryo splitting may occur through mechanisms other than complete physical separation of an early embryo. Current models therefore place greater emphasis on cellular events occurring within the blastocyst, particularly the organization and separation of the inner cell mass (ICM) (13).

One proposed mechanism involves the formation of spatially distinct cell populations within the ICM. Experimental and clinical observations have raised the possibility that reduced cell–cell adhesion, altered ICM organization, blastocyst expansion and collapse, or mechanical forces associated with hatching may facilitate separation of pluripotent cell populations (13, 15, 16). This concept is particularly relevant to monozygotic triplet formation, for which a single splitting event is insufficient to explain the development of three embryonic axes. Sequential splitting or the establishment of more than two independent ICM cell populations has therefore been proposed, although direct evidence for either mechanism in human embryos remains lacking (13).

The ultrasound timeline should be interpreted as the timing of clinical recognition rather than the timing of embryo splitting. The first examination, performed 21 days after embryo transfer, showed a singleton intrauterine pregnancy, whereas three viable embryos were identified at 8 weeks and 1 day. This interval cannot establish that splitting occurred between the two examinations, because the embryological event would have preceded sonographic recognition and early ultrasound may not identify the final number of embryos or amniotic compartments. Accordingly, the sequence of ultrasound findings provides no direct evidence regarding when splitting occurred.

The present case cannot establish when or why embryo splitting occurred. Monozygotic triplet formation occurred despite the absence of assisted hatching and trophectoderm biopsy, showing that these interventions are not prerequisites for this outcome. The blastocyst also underwent marked post-warming expansion from grade 2BB to 6BB before transfer. Whether spontaneous hatching, post-warming dynamics, or day-6 development had any relationship to subsequent splitting cannot be determined from a descriptive case, and no causal inference should be made. The value of the present report therefore lies in documenting an uncommon combination of embryological characteristics and clinical outcome that can be compared with other rare cases rather than in providing mechanistic evidence.

## Conclusion

Single embryo transfer remains the preferred strategy for minimizing iatrogenic multiple pregnancy and maximizing the likelihood of singleton birth. This case documents the exceptionally rare occurrence of a monochorionic triamniotic triplet pregnancy after transfer of a single day-6 vitrified-warmed blastocyst in the absence of assisted hatching or trophectoderm biopsy. Together with the limited published cases, it illustrates the heterogeneity of ART characteristics associated with monozygotic triplet pregnancies without establishing a specific mechanism of embryo splitting.

## Data Availability

The original contributions presented in the study are included in the article/Supplementary Material, further inquiries can be directed to the corresponding author.
